# Adaptive-Driven CT Simulation-Free Soft Tissue Stereotactic Body Radiation Therapy: A Single-Patient Case Report

**DOI:** 10.7759/cureus.66876

**Published:** 2024-08-14

**Authors:** Malorie R Veres, Yasamin Sharifzadeh, James A Kavanaugh, Sean Park, Victor Malkov

**Affiliations:** 1 Radiation Oncology, Mayo Clinic, Rochester, USA

**Keywords:** sbrt, simulation-free, hypersight, adaptive, ethos

## Abstract

Online adaptive radiotherapy (ART) enables accommodation for variations in patient setup and anatomical changes, allowing for fractional replanning for target coverage, organ at risk (OAR) sparing, and application of CT simulation-free (SF) workflows. SF workflows bypass the conventional simulation CT scan at the potential trade-off in dosimetric uncertainty. ART can alleviate many of these uncertainties, and this work extends previous experience with an Ethos adaptive cone-beam computed tomography (CBCT)-based SF process to treating a unique bony and soft tissue case with stereotactic body radiation therapy (SBRT).

The patient is an 83-year-old male with metastatic prostate cancer, presenting with metastases near the right posterior ischium and a right perirectal lymph node. The patient's history includes multiple radiation treatments and androgen deprivation therapy (ADT). Rising prostate-specific antigen(PSA) levels and new metastases identified via positron emission tomography (PET)/CT prostate-specific membrane antigen (PSMA) led to SBRT re-irradiation, considered safe due to the time lapse since previous treatments. Using a HyperSight-equipped Ethos ART system, an SF SBRT workflow utilized the patient's recent PET/CT images for target and OAR delineation. A nine-field adaptive intensity-modulated radiotherapy(IMRT) treatment plan was generated to deliver 3600 Gy in three fractions with a primary focus to limit the dose to proximal OARs and the previously treated region. At the adaptive treatment, the patient is positioned based on anatomical marks, and axial images from HyperSight CBCT are used to contour the OARs and targets. These modified contours accommodate daily variations and are used to recalculate the reference plan and generate a new adapted plan. The adapted plan is selected if coverage improvement and OAR sparing are achieved. For each newly adapted plan, Ethos-generated synthetic CT is reviewed prior to treatment to verify no errors occurred in the deformable propagation between the reference image and the fractional CBCT. For this patient, the adapted plan was selected for all fractions due to improved target coverage, particularly of the soft tissue target, and OAR sparing. The patient tolerated the treatment well and demonstrated a good response on three-month follow-up PSMA PET/CT imaging.

This case highlights the efficacy of CBCT-driven SF ART in complex re-irradiation scenario. Future enhancements in the Ethos treatment planning system, including direct dose computation on HyperSight CBCT images, will streamline SF workflows and expand their applicability. Careful consideration of potential on-unit OAR changes and target motion remains crucial for successful SF ART applications.

## Introduction

The primary goal of online adaptive radiotherapy (ART) systems is to enable fractional treatment plan optimization to account for patient setup and anatomical changes from the reference CT or MR simulation. Compared to the standard radiotherapy process, ART provides an opportunity for target margin reduction and healthy tissue sparing with the aim to reduce toxicities while maintaining tumor control. An additional benefit of ART systems is facilitating the application of CT simulation-free (SF) workflows. Diagnostic imaging-based treatment planning can reduce patient costs and time and improve quality of life [1-3] with a potential trade-off of dosimetric uncertainty due to Hounsfield unit (HU) accuracy and patient setup variation. The latter can be addressed with ART and has been used for palliative SF workflows [4,5]. Our group has presented and validated an SF workflow applied to stereotactic body radiation therapy (SBRT) treatments with a focus on osseous metastatic targets.

SBRT is routinely used in oligometastatic and retreatment scenarios [6-8]. Target coverage may be compromised for adjacent radiosensitive organs at risk (OARs), and previous treatments can be handled by either a reduction of allowed doses to these OARs or generation avoidance structures by co-registering the previous treatment plan. ART can be advantageous in re-irradiation cases to aid in adjusting for daily changes of the relative position of the target, OARs, and previously treated regions. In this work, previous diagnostic CT-based SF experience is extended to the treatment of a unique bony and soft tissue target with a history of radiotherapy in the region using a HyperSight-equipped Ethos ART system [9,10]. This process uses a positron emission tomography (PET)/CT image for reference treatment planning, and adaptive fractions are delivered using the Ethos ART system with a dose computed on the Ethos-generated synthetic CT (sCT). Utilizing the SF process for this patient allowed for a reduction in patient travel requirements, expanded pre-planning timelines, and produced an approach to limit dose to previously treated tissues.

## Case presentation

Clinical presentation

An 83-year-old male, with a long-standing history of metastatic prostate cancer, rheumatoid arthritis, sciatica with associated imbalance, hyperlipidemia, and hypertension, presented to the radiation oncology clinic for discussion of radiation treatment to metastases in an area of soft tissue near the right posterior ischium and a right perirectal lymph node. 

The patient was initially diagnosed in 2012 with Gleason 7, prostate-specific antigen (PSA) 13.72 ng/mL, and cT2aN0M0 prostate adenocarcinoma and was treated with external beam radiation therapy to 7770 cGy in 42 fractions to the prostate fossa and six months of androgen deprivation therapy (ADT). By 2014, he had rising PSA and received salvage external beam radiation therapy to 6000 cGy in 30 fractions to elective pelvic lymph nodes with an integrated boost to 7500 cGy to metastatic left pelvic side-wall lymph nodes. Despite the initial drop in PSA post-radiation, PSA continued to rise over the next five years while he was off ADT. By 2019, PET/CT choline showed metastatic disease in the rib and a mesorectal lymph node. He restarted ADT and received palliative radiation therapy to the rib, which he tolerated well. While on ADT, his PSA became undetectable. He discontinued ADT 1.5 years later given undetectable PSA and no evidence of disease. 

**Figure 1:**
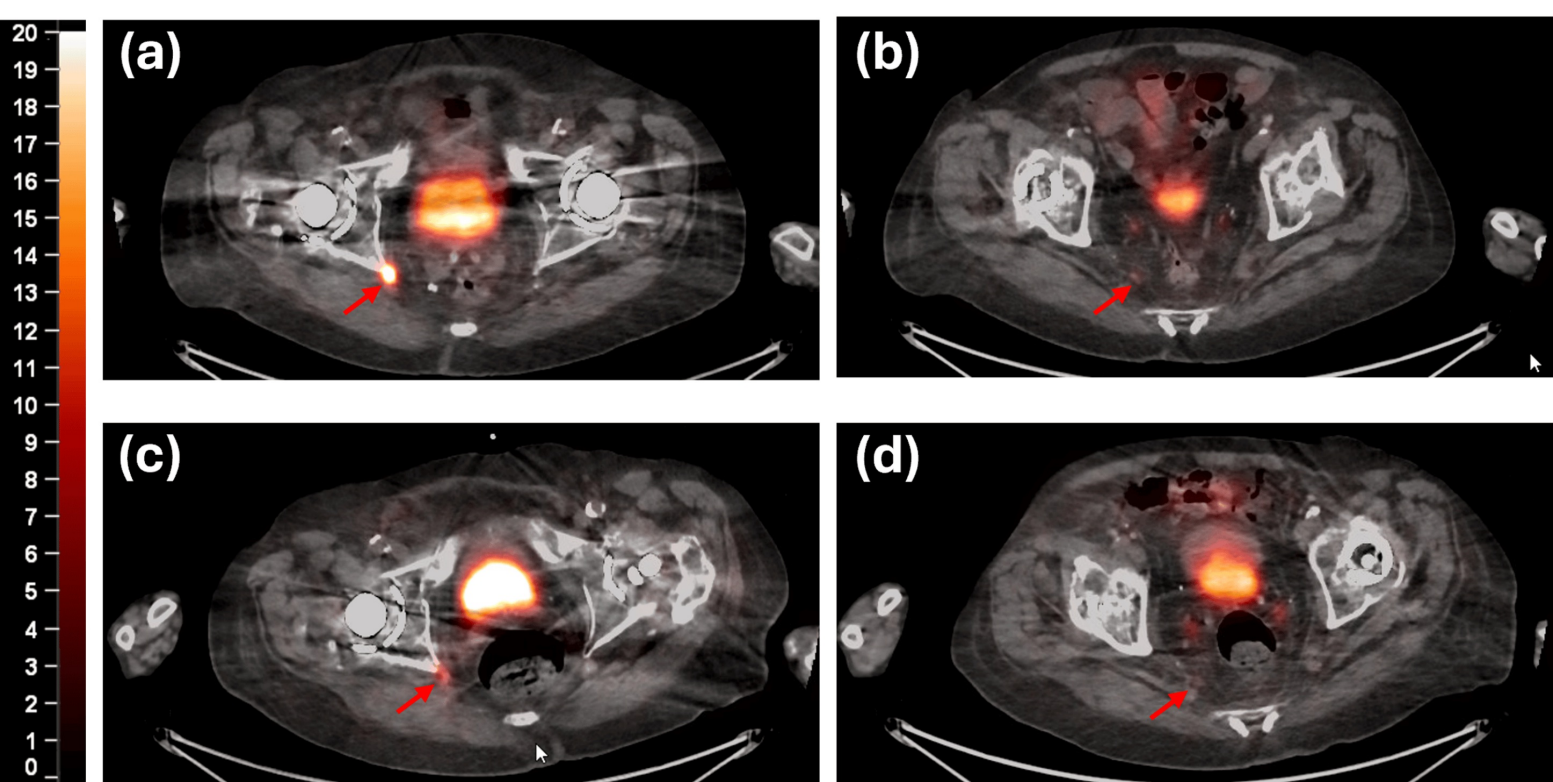
PET/CT images with SUV overlay of pre-treatment: (a) the ischium target (1.8 × 1.3 cm) and (b) the soft tissue perirectal lymph node (0.5 cm). The three-month post-treatment images are presented for (c) the ischium target and (d) the lymph node. PET: positron emission tomography; SUV: standardized uptake value

Between 2020 and mid-2022, he had slowly rising PSA without definitive abnormal uptake in serial PET/CT choline scans. However, by late 2023, his PSA had risen to 7.4 ng/mL. Prostate-specific membrane antigen (PSMA) PET/CT demonstrated focal uptake in a soft tissue mass posterior to the right posterior ischium (Figure 1a) as well as a small right perirectal node (Figure 1b). Although he endorsed some worsening of his rheumatoid arthritis, neuropathy, and imbalance, he denied new bony or pelvic pain.

Given these areas were the only sites of detectable disease, radiation therapy to both was recommended. Both areas had received radiation previously, and the risks of radiation retreatment to these areas were discussed in detail. However, given that at least 10 years had passed since conventionally fractionated radiation therapy had been delivered to both lesions, re-irradiation using SBRT was deemed a safe and effective approach. A dose of 3600 cGy in three fractions was recommended. The patient agreed to move forward with SBRT during a video virtual consultation, and given he lives four hours from the clinic, both sites were easily discernible on the CT image, and the desire to avoid the previously treated regions, the SF adaptive SBRT process was desirable. The patient consented to data usage, and this work was approved by the Mayo Clinic Institutional Review Board (IRB) (approval number: 24-003720).

Reference adaptive plan

The SF SBRT workflow consisted of using the patient's most recent PET/CT image for target and OAR delineation. The HU accuracy for dose computation was validated prior to utilizing the SF process and demonstrated HU values consistent with the radiation oncology clinical helical CT systems. The previous treatment area was delineated to include the seminal vesicles and an extended region surrounding the superior part of the prostate (prev. irradiated tissue). The CT image and contours were transferred into the Ethos treatment planning system (TPS), and a lateralized nine-field intensity-modulated radiotherapy (IMRT) adaptive treatment plan was established followed by physician/physics review and patient-specific QA. The reference plan metrics are presented in Table 1 (reported as dose per fraction) with near max OAR metrics set as priority clinical objective. A single treatment plan was created for treating both the ischium target (clinical target volume (CTV)_Bone) and the perirectal lymph node (CTV_LN). The planning target volume (PTV) was defined as a 3 mm expansion of the two targets, and an optimization PTV (PTV_opt) was defined as the PTV cropped by 2 mm from the bladder, 5 mm from the small bowel, and 6 mm from each the rectum and the previously irradiated tissue. Near-full coverage of the PTV_opt is sought with a low dose coverage requested on the PTV without conflicting with the OAR objectives.

**Table 1:** Planning dose metrics and associated reference plan, fractional scheduled (sched) and reference (ref) achieved metrics. * indicates failing dose metrics. CTV: clinical target volume; PTV: planning target volume

Structure	Metric	Target	Reference plan	Fraction 1		Fraction 2		Fraction 3	
				Scheduled	Adapt	Scheduled	Adapt	Scheduled	Adapt
CTV_Bone	V95% (%)	>100%	100	-	100	98.9*	100	100	100
CTV_LN	V95% (%)	>100%	100	-	100	69.4*	100	86.7*	100
PTV	D95% (cGy)	>167 cGy	1188	-	1200	584	1195	1019	1210
	D0.03cc (%)	<120%	118.2	-	115.9	120.4*	113.8	120.2	117
PTV_opt	V100% (%)	>95%	93.8*	-	95	70.1*	94.4*	84.3*	96.2
Bladder	D0.03cc (cGy)	<940 cGy	447	-	420	416	409	425	419
	V333cGy (cc)	<15 cc	5.67	-	1.11	3.44	1.79	2.66	1.22
Rectum	D0.03cc (cGy)	<600 cGy	471	-	323	531	349	374	291
	V300cGy (cc)	<20 cc	3.46	-	0.15	5.7	0.62	1.26	0.01
Small bowel	D0.03cc (cGy)	<500 cGy	467	-	420	509	366	537	479
	V500cGy (cc)	<5 cc	0	-	0	0.08	0	0.2	0
Prev. irradiated tissue	D0.03cc (cGy)	<217 cGy	206	-	194	239*	194	395	209

Online adaptive treatment

**Figure 2:**
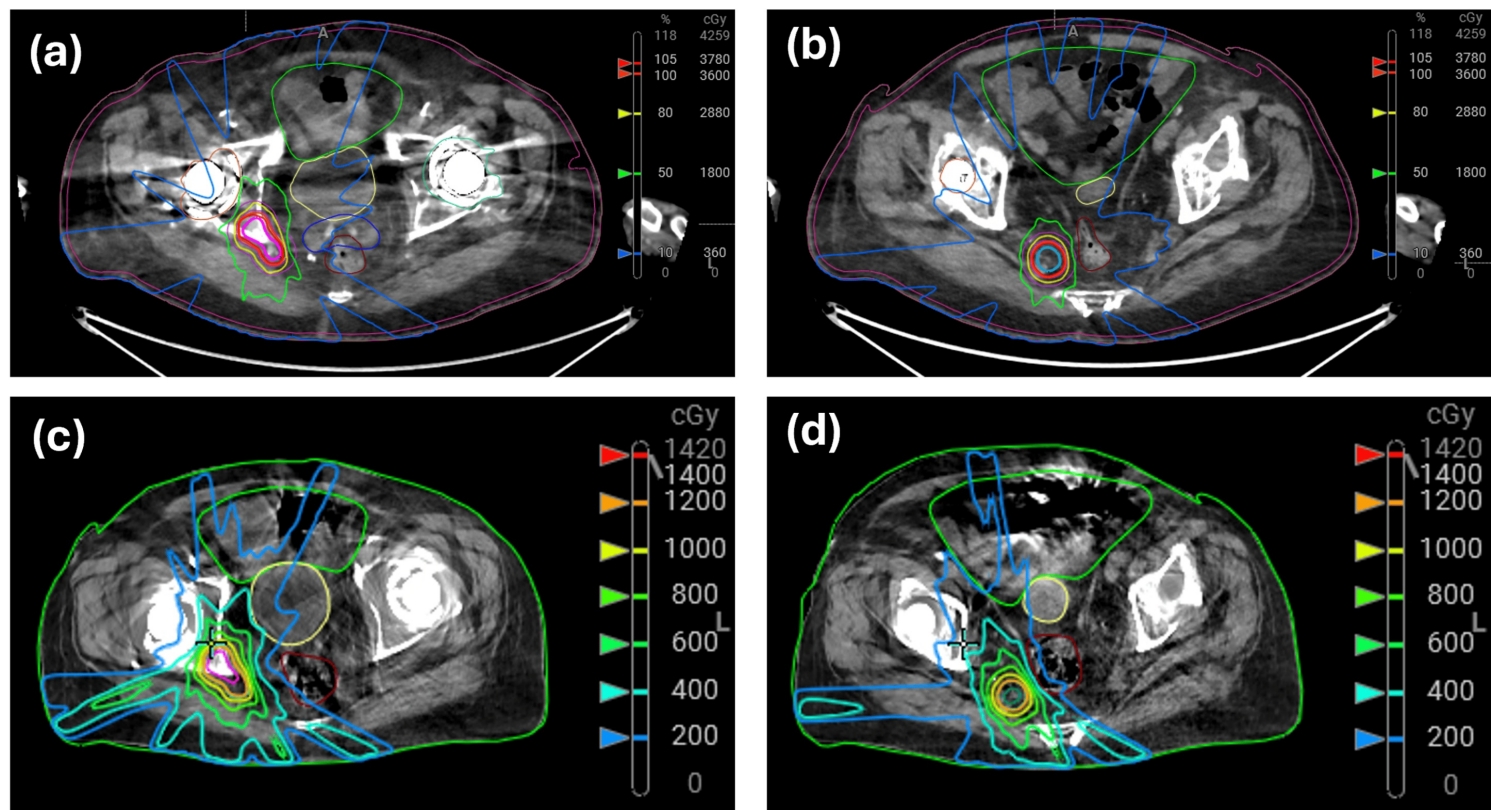
Reference adaptive treatment dose distribution for (a) CTV_Bone and (b) CTV_LN. The second fraction adapted plan dose distribution is presented for (c) CTV_Bone and (d) CTV_LN. Window = 300 HU and level = 40 HU were set for both images. CTV: clinical target volume; HU: Hounsfield unit

At the first adaptive fraction, the patient was aligned based on anatomical reference (mid-patient at umbilicus), and immobilization was created for subsequent fractions. Next, for all adaptive fractions, the adapt CBCT (pelvis large scanning protocol with Acuros/MAR reconstruction) was collected and reviewed for imaging artifacts. The Ethos-generated automated contours (influencer structures) for the bladder and rectum were reviewed and edited by a trained medical dosimetry assistant (MDA). Additionally, the automatically generated body contour was carefully inspected for irregularities. Within the Ethos contouring space, the deformed small bowel was adjusted by the MDA, and the previously irradiated tissue and the rigidly propagated CTV_Bone and CTV_LN were modified by the radiation oncologist. To optimize on-unit contouring, a 2 cm expansion from the planning PTV structures is used to help focus the region for accurate OAR delineation. These contours were forwarded to the Ethos optimizer to plan generation. The scheduled and adapted plans were reviewed by the physician and physicist with a threshold for selecting the adapted plan based on a minimal 5% target coverage improvement and target/OAR clinical goal violation. Mobius secondary dose calculation was used for pre-treatment and post-treatment log file-based QA. The Ethos sCT image was reviewed prior to treatment to capture potential deformation errors between the diagnostic reference CT and the adapt CBCT. The sCT was directly inspected in Mobius to ensure that bone components aligned with the osseous target, soft tissues were correctly mapped based on the soft tissue target and contoured OARs, and no erroneous high or low densities were mapped in or near the targets. Further, the alignment of high-density mapping to match bony anatomy was reviewed in Ethos. The comparison of scheduled and adapted plan metrics is presented in Table 1, and a sample dose distribution for the two targets is shown in Figure 2c-2d. The 2 cm guidance contour was set up as a target with a low-dose prescription, and at the first fraction, due to position and anatomical changes, 150% of this dose fell outside this structure for the reference (scheduled) plan. This triggered an automated internal Ethos dose limit rule which caused the scheduled plan to not be computed and is unrelated to the use of the SF process. The first fraction metrics would have resulted in similar dose coverage values to the latter fraction based on a computation of the dose on the adapt structures and CBCT within the Eclipse TPS. The adapted plan was selected for all fractions, driven primarily by target coverage and sparing achieved in the previously irradiated tissue region. Following treatment plan selection, a verification CBCT is collected to rigidly adjust for patient position changes and confirm no substantial target/OAR motion occurred.

The time per fraction is provided in Table 2. This data was extracted from the Digital Imaging and Communications in Medicine (DICOM) time stamps from each of the fractions. The setup time encompasses the time from when the patient is opened on the unit to the collection of the planning CBCT image, the total adapt time ranges from the planning CBCT collection through the treatment end, the contouring time is from the planning CBCT collection through the start of plan computation and optimization, and the plan review time follows plan creation to the verification CBCT collection.

**Table 2:** Time distribution per fraction, for initial patient setup, total active adaptation, contouring, and plan review times.

Fraction	Setup time (min)	Total adapt time (min)	Contouring (min)	Plan review (min)
1	22	40	23	5.0
2	7	35	18	3.2
3	11	30	14	2.25

## Discussion

As shown in Table 1, the adaptive plans generated an improvement in target coverage, mainly for the soft tissue CTV_LN target, and a reduction of the dose to the previously irradiated soft tissue. Though the bladder, rectum, and small bowel structures did not exceed the clinical goals on the scheduled plans, the adaptive plan accommodated for per fraction anatomy and reduced both near max and volume metrics. The active contouring time (from initial CBCT acquisition to treatment completion) ranged from 30 to 40 minutes with the initial fraction taking being the longest. The patient was able to tolerate the treatment well and showed a good response in the treated region (Figure 1c-1d) based on three-month follow-up imaging.

Traditional CBCT imaging is limited for use in adaptive radiotherapy due to field size and image quality limitations [11,12]. Particularly bilateral hip prosthesis is commonly a challenge for CBCT imaging systems. The HyperSight imaging system provides a reduction in image artifacts and an improvement in HU accuracy [13]. Figure 2c-2d demonstrates the capability of the HyperSight system in being able to produce CBCT images with limited imaging artifacts for the SF patient with bilateral hip prosthesis. Further upgrades to the Ethos TPS, allowing for direct dose computation on HyperSight CBCT (Acuros/MAR reconstruction), will become available. This will provide an opportunity to reduce the need for detailed HU validation of the diagnostic CT images and simplify beam geometry selection during reference adaptive planning. The concern of errors in the sCT generation due to substantial patient setup differences between the diagnostic CT and on-unit setup would be eliminated, which results in the major advantage of utilizing a much broader range of sources for reference diagnostic CT images, even those with a limited field of view. 

In the SF process, a careful review of the reference images is currently required to provide a reliable dose distribution on the unit. Some of these considerations include artifacts in the diagnostic CT, using a lateralized beam to avoid entry near the limit of the field of view of the CBCT, limiting the external contour to accommodate for on-unit setup (e.g., arms up vs down), and others. As noted above, direct on-CBCT dose computation will reduce many of these considerations but will also require treatment planning which accommodates for potential OARs which may not be present on the diagnostic image. This can be either due to a change in patient anatomy/setup or if using a limited field-of-view diagnostic CT image. Further, regardless of on-CBCT dose computation, the slice thickness of the reference diagnostic image and the HyperSight CBCT (2 mm) needs to be considered when contouring targets on the order of this thickness in the superior/inferior direction. Diagnostic images do not provide target and OAR motion assessment and as such may limit application to targets expected to have substantial motion (abdominal). Breath-hold for imaging and treatment during the adaptive fraction may be an alternative for these cases but presents the challenge of patient breath-hold compliance assessment or stability/reproducibility prior to the patient treatment. Certain sites are well suited for SF application, and others may require further technique development or should be directed through conventional simulation CT pathways.

## Conclusions

This work presents a unique case of the treatment of a soft tissue target using a CBCT-driven SF process with SBRT. Adapted treatments provided consistent coverage and OAR sparing improvement at all fractions without notable errors in the sCT generation. Future upgrades to the Ethos TPS allowing for direct dose computation on HyperSight CBCT images will notably ease the barrier of SF workflow implementations. Careful forethought of possible on-unit OAR geometry and target motion would remain essential.
